# Detection of schistosome cercariae in water: A systematic review of traps and attractants

**DOI:** 10.1371/journal.pntd.0014625

**Published:** 2026-08-17

**Authors:** Amelia Rupp, Michael R. Templeton, Laure Sioné, Ana Pitol-Garcia, Laura Braun

**Affiliations:** 1 Department of Civil and Environmental Engineering, South Kensington Campus, Imperial College London, London, United Kingdom; 2 Liverpool School of Tropical Medicine, Departments of Vector Biology and Tropical Disease Biology, Centre for Neglected Tropical Diseases, Liverpool, United Kingdom; 3 Department of Disease Control, Faculty of Infectious and Tropical Diseases, London School of Hygiene and Tropical Medicine, London, United Kingdom; Jiangsu Institute of Parasitic Diseases, CHINA

## Abstract

Environmental surveillance of schistosome cercariae concentrations in water bodies constitutes an important complementary indicator to human schistosomiasis infection data. *Schistosoma* cercariae traps can be one means of environmental surveillance. To advance these cercariae trap technologies, it is first necessary to synthesise the existing evidence on schistosome cercariae attractants and traps. Six databases were last searched in April 2025. Studies were screened to include primary research on schistosome cercariae traps and attractants with quantitative outcomes, excluding studies assessing kinesis or stages other than attraction. Results were narratively synthesised due to methodological heterogeneity. A bespoke tool was used to assess methodological quality. A total of 1951 papers were found across six online databases. The broad search string retrieved many papers that were deemed to be outside the scope of the review, meaning only eleven studies were included in this review. The attractants included light, temperature, natural oils, beeswax, fatty acids, and pifithrin. Eight studies tested traps on *S. mansoni*, one studied *S. japonicum*, one studied both *S. japonicum* and *S. mansoni,* and one studied S*. haematobium*. For *S. mansoni*, based on two studies, light was reportedly the most effective attractant, with cercariae capture percentages of 87–98%. Based on the limited evidence, beeswax alone was reportedly the least effective, giving cercariae capture percentages of 0.0-9.2%. Based on two studies, *S. japonicum* cercariae showed a greater attraction to chemicals than *S. mansoni*, with capture percentages as high as 83.2%. Future studies should focus on consistency to allow comparability, whilst increasing the variety of schistosome species studied and testing new trap designs. This study can therefore guide the development and deployment of cercariae traps to provide insight into the contamination status of water bodies. This will serve as a useful tool in monitoring the progress towards the elimination of schistosomiasis as a public health concern.

## Author summary

Environmental detection of schistosome cercariae could provide a complementary method to human diagnostic testing and provide a greater understanding of the impact of preventive measures and mass drug administration on the residual contamination in water bodies and the risk of re-infection. Cercariae traps could, in theory, provide a lower-cost, scalable option which is able to provide continuous, real-time data on cercariae concentration in water bodies. This systematic review aims to summarise and synthesise the existing research into schistosome cercariae traps and attractants to identify opportunities for future research. This review can therefore guide future development of traps, which can be valuable complementary tools in support of the effort to eliminate schistosomiasis.

## Introduction

Schistosomiasis is a water-based neglected tropical disease (NTD) caused by parasitic blood flukes. Over 200 million people worldwide are estimated to be infected, with over 700 million people living at high-risk of infection [1]. As part of the World Health Organization’s Roadmap to Ending NTDs, it is targeted for elimination as a public health problem, defined as less than 1% of the population suffering from heavy intensity schistosomiasis infections, by 2030 [2]. Schistosomiasis is a disabling disease, leading to approximately 3.3 million daily adjusted life years (DALYs) (2021) [3]. Of the 23 *Schistosoma* species, five are approximated to be responsible for 80% of human schistosomiasis: *S. mansoni*, *S. haematobium*, *S. japonicum*, *S. intercalatum*, and *S. mekongi* [3].

Schistosomiasis is contracted through contact with freshwater bodies contaminated with schistosome cercariae [4]. After excretion from the definitive host and once in a water body, *Schistosoma* eggs hatch and release miracidia, which infect the intermediate snail host [5]. The snail then releases cercariae, the infective stage of schistosomiasis, which penetrate the definitive host’s skin [5].

Cercariae are the larval stage of trematodes, including schistosomes. Schistosome cercariae are approximately 500 µm in length [6], are free-swimming, and move at a mean speed of 1.29 mm/s [7]. They can survive for up to three days outside of their snail host, and are non-feeding, relying on endogenous glycogen reserves which deplete rapidly [8]. Schistosome cercariae can be thermotactic (attracted to temperature) [9], chemotactic (attracted to chemicals) [10,11], and phototactic (attracted to light changes) [12] to help them find a host.

Currently, the three principal methods of detecting cercariae in water are the use of sentinel animals, classical cercariometry, and, more recently, environmental DNA (eDNA) detection.

The use of sentinel animals involves containing rodents in cages and floating them in the test water for a few hours [13]. After removal from the water, the rodents are kept alive for at least six weeks before being dissected to check whether adult schistosome worms are present [14–16]. As well as the problem of the long timeframe of this method, and its associated costs [17], the ethics of using animals must be considered. Furthermore, it can only be used as a presence/absence test [13] as there is no linear relationship between worm load and cercariae concentration [17].

Classical cercariometry refers to the quantification of cercariae in the water without using molecular methods. The most common method is to filter a water sample, stain the filter, and count the cercariae by microscopy [13]. Centrifugation has also been used to pull cercariae to the bottom of a water sample, where they can then be counted using microscopy [13]. Whilst both these methods allow the cercariae concentration to be quantified directly across time and space, the efficacy of cercariae capture is noticeably reduced with increased turbidity [18], trained personnel are required to identify the cercariae based on morphology, its sensitivity is low, and it can be laborious [17]. Even with trained personnel, the difficulty of differentiating between cercariae species is a limitation of this method, and morphology can only identify cercariae to family level [13,14]. For species-specific identification, molecular tools, such as PCR, are required [13].

Detection of DNA from the environment also requires an initial filter stage in which a water sample is filtered to remove particulate matter while retaining eDNA. The filter is then processed using various chemical extraction protocols to isolate the target DNA, in this case, specified species of schistosome cercariae [17]. The extracted DNA is then analysed using molecular detection methods, commonly quantitative PCR in schistosome eDNA studies [17]. Whilst eDNA-based molecular detection can have high specificity and sensitivity [19], it cannot differentiate between dead and alive cercariae, has a high cost, requires a suitably equipped laboratory environment and trained professionals [20–22] and can cause false negatives if the DNA levels are too low [23].

Conversely, cercariae traps have the potential to be low-cost, locally accessible methods for quantifying cercariae [22]. Traps can be broadly categorised based on their mode of action; passive traps collect organisms that incidentally encounter them [24], baited traps actively attract cercariae [10], and attractant-based traps using chemically synthesised cues, which represent a subset of baited traps [22]. Baited traps exploit the chemotactic, phototactic, and thermotactic behaviour of cercariae.

Monitoring schistosome cercariae provides a direct indicator of active transmission, as the presence of cercariae confirms that intermediate host snails are infected and that eggs are reaching the water. This can reduce the risk of false negatives associated with snail-based assessments and human diagnostics, although no detection method is perfectly sensitive. Studies using mice have shown that higher cercarial concentrations in water bodies tended to give an increase in infection rates and mean worm burdens [25,26]. Furthermore, analysis carried out by Madubueze et al. [27] found that cercariae concentration in the water was one of the most influential factors in mathematical models, but that this factor itself contains a large uncertainty due to the complications of determining cercariae concentration in water [10,28]. The potential of traps to provide continuous monitoring of cercariae allows site- and time-specific data to be collected that can strengthen our understanding of the factors that affect cercariae concentrations. These include the periods of peak cercarial exposure, potential ecological determinants of cercariae concentration, such as water temperature, vegetation, and turbidity, and the effects of environmental and anthropogenic interventions, such as dams, irrigation, mass drug administration, and water, sanitation and hygiene (WASH) programmes.

Despite their potential, no cercariae trap has been deployed in the field as a monitoring device. This could be due to the attractants tested, the sensitivity of the traps using these attractants, or the limited data collected from field-based studies. Therefore, summarising the existing evidence on traps and attractants is essential to identify approaches with potential for field deployment and to inform future potential research and development pathways.

In the wider context of current S*chistosoma* detection methods, the primary focus is on the prevalence and intensity of infection in human hosts, measured using diagnostic tests analysing urine, stool or blood, such as Kato-Katz, serological or immunological tests. The results of these tests often influence the treatment and support a community receives, particularly in guiding the distribution of preventive chemotherapy programmes [29,30].

Methods considered less expensive and easier to implement in the field, such as egg detection techniques like Kato-Katz and urine filtration, have low sensitivity, particularly in low-to-moderate intensity of infection cases [31–33]. This is due to heterogeneous egg distribution and temporal variability in egg excretion [33]. Additionally, these methods require microscopy training and are labour-intensive [34]. Once these limitations are considered together, particularly the personnel cost [18], the true cost is higher than initially calculated, they are less implementable [34], and budget constraints require a compromise between sensitivity (the number of stool samples per person) and statistical precision (the number of people surveyed) [35]. Furthermore, the false negatives can lead to an underestimation of infection numbers within a community [36]. This can be impactful in deciding when to stop preventive chemotherapy programmes, as this is when the infection intensity will be low [35]. Conversely, molecular testing is more accurate and has a higher sensitivity, but it requires specialised equipment, further training, cannot differentiate between active infection and residual DNA, and is vulnerable to contamination causing false positives [33]. In addition to these challenges, all sampling methods require consideration of ethics, particularly as children and vulnerable populations may be involved, which can lead to additional cost, time and labour [37].

Environmental factors could contribute to a more accurate understanding of schistosomiasis ‘hotspots’ (communities with infection prevalence “≥ 10% that demonstrate lack of an appropriate response to two annual rounds of preventive chemotherapy”) [30]. However, the existing environmental methods also have challenges. Snail monitoring is a widely used environmental detection method, but it has a high labour and time cost, requiring trained personnel to manually collect snails from extensive survey areas [38]. Furthermore, it has a low sensitivity as often only a few percent of the snails collected are found to be infected [38] and there are temporal variabilities [13]. After collection, the snails are encouraged to shed cercariae or are dissected, and the cercariae are viewed using microscopy [39]. Alternatively, molecular methods, such as amplifying the schistosome DNA within the snail or performing tests on the snail to identify specific antibodies, can be employed to determine whether the snail is infected [39]. This can take time and often means snails must be transported to facilities that can support this work. Microscopy methods have the risk of producing false negatives if the infection intensity in the snail is low or the infection is non-patent or pre-patent [40]. Whilst molecular methods do have higher sensitivity, they require an uncontaminated laboratory environment and expensive materials.

This systematic review aimed to summarise and synthesise the current evidence on the efficacy of schistosome cercariae attractants and traps and identify key knowledge gaps. This will be useful to guide future research and development of traps and thereby support efforts to move towards the elimination of schistosomiasis as a public health concern.

## Methods

Six electronic databases were searched: Google Scholar, PubMed, Web of Science, Scopus, Embase, and ProQuest. ProQuest was used to search for solely grey literature, whereas the others had no limitation in this regard. Publication dates were not restricted, meaning searches were conducted on literature of any date until April 2025, when the search was performed. Additional sources included manual screening of the reference lists of studies included in this review and of papers classified as ‘similar’ on the webpages of the included studies.

Search strings were created for each electronic database using a Boolean strategy with a combination of terms in four categories: Schistosomiasis, cercariae, traps and attractants, and measurement methods. The basic search string was as follows: (schistosom* OR bilharz*) AND (cercaria*) AND (trap* OR attract* OR bait* OR “chemical cue*” OR “host cue*” OR lure*) AND (detect* OR monitor* OR quantif* OR surve* OR sampl* OR collect*). The asterisks indicate where truncation was used (e.g., schistosom* retrieved schistosomiasis, schistosome, schistosoma and related word variants). The search terms and the search string for each database can be found in S1 Text.

All retrieved records were imported into a reference software, EndNote, and de-duplicated automatically. The resulting records were then uploaded onto Rayyan where remaining duplicates were removed using a combination of automated detection and manual review. Using the pre-defined exclusion and inclusion criteria (supplied in S2 Text), the de-duplicated set was screened independently by two reviewers, first by title and abstract and then by full text (AR, LS). Records were screened by title and abstract in one stage to ensure that they were not discounted if the title was misleading. No restrictions were placed on language, document type or date. Both grey literature, such as non-peer-reviewed theses, and peer-reviewed publications could be included, if they fit the other criteria. For studies published in languages other than English, Google Translate was used and the translation was checked with native speakers. Studies were excluded based on the following criteria: study type (background or commentary articles and non-original research); topic scope (research relating to non-schistosome cercariae, cercarial penetration, attachment, or creep – defined as movement of cercariae across the skin to locate a penetration site – and kinesis rather than taxis); and reported outcomes (qualitative-only results with no quantitative data). The focus on kinesis rather than taxis is demonstrated throughout this review with the use of the word ‘attractant’, implying that the cercariae actively move towards the source, showing tactic behaviour. Any use of the word ‘stimulant’ is used to refer to a source that causes a change in the movement of the cercariae, indicating kinetic behaviour.

Where possible, authors or their related institutions were contacted where papers were unretrievable online. Discrepancies were resolved through discussion. The full selection process with the associated numbers of papers is discussed in the Method.

Data was extracted manually and input into a data extraction sheet by AR in June 2025. Due to substantial heterogeneity in experimental designs, exposure conditions and result reporting, a narrative synthesis was used with findings grouped thematically (e.g., by type of attractant, species of *Schistosoma*, and experimental setting).

In order to compare the efficacy of attractants quantitatively, two values were calculated: an efficacy ratio and a cercariae capture percentage. The efficacy ratio compares the number of cercariae captured when the attractant is applied (‘treatment efficacy’) to when only a control is used, and the capture percentage evaluates the percentage of cercariae that were trapped. The general formulae for the values are shown below, although some adjustment between the studies was required.


$$\begin{array}{c} Efficacy\;ratio=\;\frac{treatment\;efficacy}{control\;efficacy} \end{array}$$

$$\begin{array}{c} Capture\;\%=\text{100}\times \frac{\left(number\;of\;slides\;\times number\;of\;cercariae\;captured\;per\;slide\right)}{total\;number\;of\;cercariae} \end{array}$$

For example, if a paper reported the capture percentage as 56.3% when the attractant was applied and 7.9% in the control scenario, the efficacy ratio would be calculated as:


$$\begin{array}{c} Efficacy\;ratio=\;\frac{\text{5}\text{6}.\text{3}}{\text{7}.\text{9}}=\text{7}.\text{1} \end{array}$$

Alternatively, if a paper reported the average absolute number of cercariae captured per slide as 121.7, the number of cercariae as 61,000, and the number of slides as 50, the capture percentage would be calculated as:


$$\begin{array}{c} Capture\;\%=\text{100}\times \frac{\left(\text{5}\text{0}\;\times \text{1}\text{2}\text{1}.\text{7}\right)}{\text{6}\text{1},\text{0}\text{0}\text{0}}=\text{1}\text{0}.\text{0}\% \end{array}$$

If the volume of water was not reported in the paper, the volume was calculated using the given dimensions of the container used.

The study by Shiff et al. in 1994 [11] recorded the attractant efficacy by assessing how far the cercariae moved towards the attractant. After the test duration had elapsed, a locking system split the choice chamber containing the cercariae into 14 evenly spaced isolated compartments, trapping the cercariae that had moved as far as each compartment. Each compartment was then drained through a filter, which was stained, and the number of cercariae counted [11]. This method meant that an efficacy ratio or cercariae capture percentage could not be calculated as previously described. Instead, using data from bar graphs in the study, which was available for both the control and attractant (‘treatment’) tests, the number of cercariae in each compartment and their distance travelled was combined to find a dispersal score. The control and attractant (‘treatment’) dispersal scores were then compared to find the efficacy ratio.


$$\begin{array}{c} Dispersal\;score=\frac{\sum_{n=\text{1}}^{\text{1}\text{4}}\left(number\;of\;cercariae\;in\;compartment\;n\times distance\;to\;compartment\;n\right)}{total\;number\;of\;cercariae} \end{array}$$

$$\begin{array}{c} Efficacy\;ratio=\;\frac{treatment\;dispersal\;score}{control\;dispersal\;score} \end{array}$$

Cohen, Neimark & Eveland [9], who investigated the effect of heat on *S. mansoni*, recorded their results as cercariae per millilitre in the heated and unheated arms of a U-tube. A value for the control concentration was not given. However, the paper states that the control test resulted in no change in cercariae concentration in either of the U-tube arms. Using the provided values for the volume of water used and the number of cercariae, an average concentration of cercariae was calculated and used as the control efficacy value.


$$\begin{array}{c} Control\;cercariae\;concentration=\;\frac{total\;number\;of\;cercariae}{water\;volume} \end{array}$$

$$\begin{array}{c} Efficacy\;ratio=\;\frac{heated\;arm\;cercariae\;concentration}{control\;cercariae\;concentration} \end{array}$$

The quality of the studies was also assessed using an evidence level system created specifically for this review. This system assessed the studies on the completeness of experimental information reported, such as attractant information, cercariae information, and experimental conditions, as defined in S3 Text.

## Results

**Fig 1:**
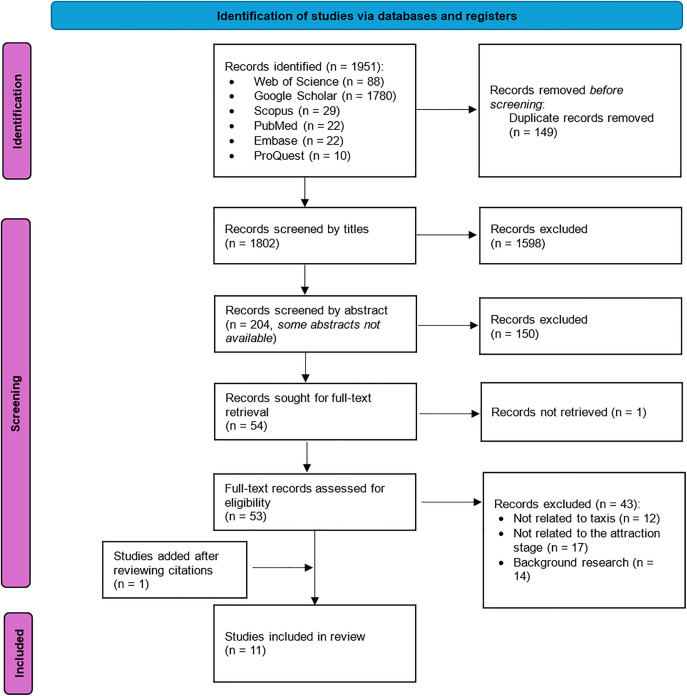
PRISMA flow diagram.

Across the six databases searched, a total of 1951 records were retrieved (see PRISMA flow, Fig 1). Of these, 149 were duplicates, leaving 1802 to be screened by two independent reviewers. Due to the encompassing search string, many irrelevant records were retrieved, leading to many being excluded at the first screening stage. After screening by title and abstract, 54 records remained. Many of these were excluded as they did not relate to schistosome cercariae, they studied human diagnostics, snail surveys, or infrastructure projects, or they were general overviews or commentaries of various parasites and environments. The full-text of these 54 records were screened, of which one was not retrieved, leading to 53 full-text records being assessed for eligibility. The paper that could not be retrieved was a thesis entitled ‘Observations on Naturally infected *Bulinus Truncatus* and *Biomphalaria pfeifferi* snails and efficiency of honey-wax cercarial-recovery trap’ [41]. 43 records were excluded at the full-text stage, leaving ten to be included. Of the 33 records excluded at this stage, 14 were excluded as they did not study the attractant stage of the cercariae’s interaction with the definitive host. Instead, they studied the attachment, creep, or penetration stage or kinesis. Other exclusion reasons were because no data, qualitative or quantitative, was included, and they were not laboratory or field-based studies. After reviewing the reference lists of each of the ten included records, one record was added, leading to a final number of eleven records included in this systematic review, shown in Table 1.

**Table 1:** Summary of included papers.

Study	Year	Attractant(s) Studied	Mechanism of Attraction	*Schistosoma* Species	Experimental Setting
Klock [12]	1961	Light	Phototaxis	*mansoni*	Laboratory and field-based
Sandt [18]	1972	Light	Phototaxis	*mansoni*	Laboratory only
Cohen, Neimark & Eveland [9]	1980	Temperature	Thermotaxis	*mansoni*	Laboratory only
Shiff et al. [24]	1993	Linoleic acid	Chemotaxis	*mansoni*	Laboratory and field-based
Shiff & Graczyk [11]	1994	Linoleic acid	Chemotaxis	*mansoni*	Laboratory only
Ahmed et al. [10]	2002	Linoleic acid; sesame and olive oil mixture on wax	Chemotaxis	*mansoni*	Laboratory and field-based
Allah & Ahmed [42]	2011	Beeswax; sesame oil on wax	Chemotaxis	*haematobium*	Laboratory only
Lee et al. [43]	2013	Beeswax; oleic acid; varnish	Chemotaxis	*mansoni*	Laboratory only
Nobuo [44]	2013	Linoleic acid; stearic acid; oleic acid	Chemotaxis	*mansoni*	Laboratory only
Lee [45]	2017	Beeswax; beeswax, oleic acid and ‘hand-warmer’	Chemotaxis; combination	*mansoni and japonicum*	Laboratory only
Abellaneda et al. [22]	2024	Beeswax; beeswax and pifithrin-µ; beeswax and olive oil	Chemotaxis	*japonicum*	Laboratory only

**Fig 2:**
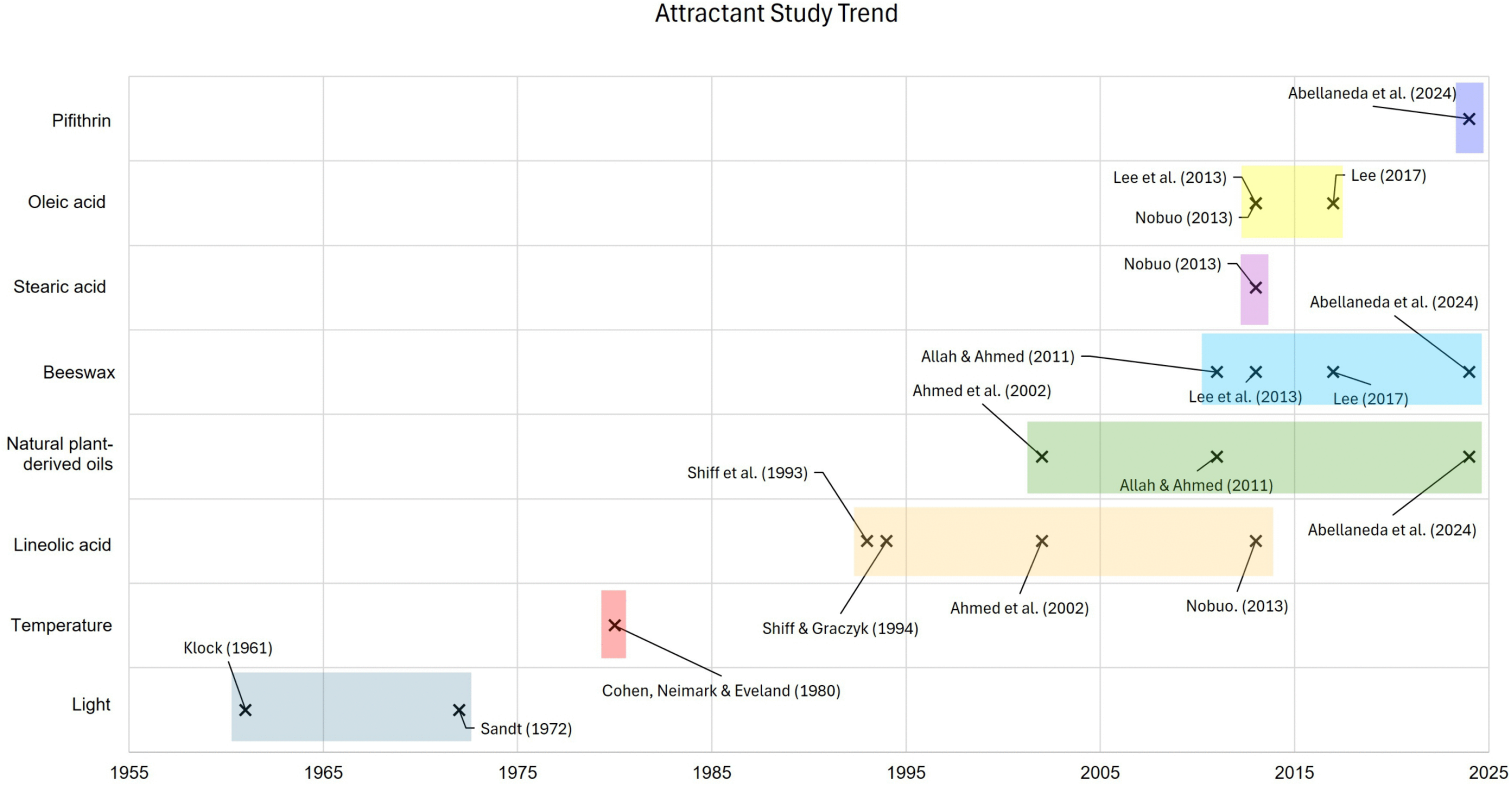
Attractants studied since 1961.

Of the eleven studies included in this review, eight studied *S. mansoni* [9–12,18,24,43,44], one studied *S. japonicum* [22], and one studied both *S. japonicum* and *S. mansoni* [45]. One did not directly specify the species, instead describing the species as ‘Human’ *Schistosoma* cercariae [42]. However, this record specifies the snail species used to shed the cercariae as *Bulinus truncatus*. This snail species and the *Schistosoma* species description lead to the conclusion that the *Schistosoma* cercariae used were *S. haematobium.* Placing the included studies on a timeline (Fig 2) suggests that there has been a more consistent focus on chemical attractants historically.

Whilst some papers reported their results using a ratio, others did not, including Klock [12] and Sandt [18], who recorded their results as percentages with no control value listed. Thus, an efficacy ratio could not always be calculated. Furthermore, the varied volumetric and environmental factors across the studies meant direct quantitative cross-comparison was often not possible.

Of the attractants tested, for *S. mansoni*, the limited evidence suggests that light was the most effective, with Klock [12] and Sandt [18] reporting capture percentages of 98% and 87%, respectively. Heat was reported to be somewhat effective with efficacy ratios ranging from 2.1 to 8.3 [9]. The chemical attractants appeared to be far less effective with linoleic acid giving the highest capture percentage of 59.8% [10]. The lowest mean capture percentage was 2.4% when testing beeswax alone [43]. The addition of 0.3 g/ml oleic acid increased the mean capture rate to 15.2%, representing the largest observed increase (531%) relative to beeswax alone [43].

For *S. japonicum*, light and heat were not tested as attractants. However, based on two studies, they showed a much greater sensitivity to beeswax, with a capture percentage of up to 80.9% [22]. This increased to 83.2% with the addition of 0.09 mg/ml of pifithrin [22]. The addition of 0.52 g/l of olive oil made little difference [22]. The addition of oleic acid caused a mean capture percentage reduction of greater than 59% compared to beeswax on its own [45]. Linoleic acid was not tested on *S. japonicum* [45].

One of the factors that changed within and across studies was the volume of water used. Volumes varied from petri dishes containing approximately 20 ml of water [22,42,45] to canals with 2100 L [10]. Ahmed et al. [10] concluded that as the water volume increased, the effectiveness of the attractants tended to reduce. However, this is not a proportional relationship as demonstrated in Ahmed et al.’s [10] results where a 4200-fold increase in water volume led to a 7.6-fold decrease in capture percentage. Similarly, Shiff et al. [24] found a 10,000-fold increase in water volume led to a 49.3-fold decrease in capture percentage.

Two studies [10,24] tested the impact of the number of cercariae on attractant efficacy. One found a decrease in the number of cercariae led to an increase in the efficacy ratio of sesame oil and wax but a decrease in the efficacy of linoleic acid [10]. The other found that a decrease in the number of cercariae led to an increase in the efficacy of linoleic acid, although not linearly [24].

These results are summarised in Table 2. The studies conducted by Shiff et al. [24] and Ahmed et al. [10] included 13 and 26 experimental conditions respectively. Thus, only a subset of their results is shown in this table, with the remainder shown in S1 Table. The results shown in Table 2 were chosen to demonstrate the variation in the volumes used for the experiment. The results obtained from the smallest and largest volumes for each of the two studies are included. For Shiff et al. [24], a tank-based experiment is also shown to demonstrate the efficacy at a larger laboratory scale. For the study by Ahmed at al. [10], one experiment from each type of container (dish, aquarium, pond and canal) and for each chemical tested (linoleic acid and sesame oil) is shown.

**Table 2:** Attractant efficacy ratios and capture percentages.

Paper	Species	Attractant	Water Volume (mL)	Number of Cercariae	Cercariae/L	Efficacy Ratio	Capture %
Klock (1961) [12]	*S. mansoni*	Light (600 W, 110 V)	2000	–	–	–	98
Sandt (1972) [18]	*S. mansoni*	Light	–	–	–	–	87
Cohen, Neimark & Eveland (1980) [9]	*S. mansoni*	Heat (29–34 °C)	32	50,000–75,000	1,562,500*–2,340,000*	2.1*–8.3*	–
Shiff et al. (1993) [24]^b^	*S. mansoni*	Linoleic acid (4.5 mg/mL)	50 61,000 500,000	50 30,500; 61,000 60,000	1,000* 500*; 1,000*	3.1* 5.8*; 4.9* –	59.8* 7.2*–5.6* 0.6*
Shiff & Graczyk (1994) [11]	*S. mansoni*	Linoleic acid (0.9 g/mL)	280	1,000	3,571*	3.3*	–
Ahmed et al. (2002) [10]^b^	*S. mansoni*	Linoleic acid	500 30,000 1,200,000 2,100,000	300; 600 18,000; 36,000 12,000; 30,000 21,000; 42,000	600*; 1,200* 600*; 1,200* 10*; 25* 10*; 20*	3.0; 20.2 5.7; 6.3 2.5; 9.0 5.8; 14.2	14.5*; 35.7* 11.5*; 13.8* 3.6*; 4.2* 3.9*; 4.7*
		Sesame/olive oil	500 30,000 1,200,000 2,100,000	300; 600 18,000; 36,000 12,000; 30,000 21,000; 42,000	600*; 1,200* 600*; 1,200* 10*; 25* 10*; 20*	2.8; 10.7 4.5; 4.8 9.2; 7.7 7.8; 5.8	16.2*; 33.2* 15.6*; 14.7* 13.4*; 10.3* 6.3*; 7.4*
Allah & Ahmed (2011) [42]	*S.* *haematobium*	Wax & sesame oil	60	401	–	6.5	10.1*
Lee et al. (2013) [43]	*S. mansoni*	Glass Varnish Varnish & oleic acid (0.3 g/mL) Beeswax Beeswax & oleic acid (0.3 g/mL) Beeswax & oleic acid (0.9 g/mL) Beeswax & oleic acid (1.8 g/mL)	20 20 20 20 20 20 20	30 60 60 60 60 60 60	1,500* 3,000* 3,000* 3,000* 3,000* 3,000* 3,000*	– – – – – – –	0.0^ac^ 0.0^c^ 3.8^c^ 0.0^c^ 7.5^c^ 4.2^c^ 3.7^c^
Nobuo (2013) [44]	*S. mansoni*	Stearic acid Oleic acid Linoleic acid	– – –	– – –	– – –	1.2* 1.9.* 3.8*	18 15 42
Lee (2017) [45]	*S. japonicum*	Beeswax Beeswax & oleic acid (0.15 g/mL) Beeswax & oleic acid (0.3 g/mL) Beeswax & oleic acid (0.9 g/mL)	20 20 20 20	30 30 29 29	1,500* 1,500* 1,450* 1,450*	– 0.5 0.3 0.4	5.2^ac^ 2.1^c^ 1.0^c^ 1.3^c^
	*S. mansoni*	Beeswax Beeswax & oleic acid (0.15 g/mL) Beeswax & oleic acid (0.3 g/mL) Beeswax & oleic acid (0.9 g/mL)	20 20 20 20	110 60 110 110	5,500* 3,000* 5,500* 5,500*	– – – –	0^ac^ 1.5^c^ 6.2^c^ 3.9^c^
Abellaneda et al. (2024) [22]	*S. japonicum*	Beeswax Beeswax & pifithrin (0.09 mg/mL) Beeswax & olive oil (0.52 g/mL)	46 46 46	100 100 100	– – –	– 1.0* 1.0*	80.9^a^ 83.2 80.2

^a^Used as the control values in efficacy ratio calculations.

^b^Due to the large number of tests performed, only a selection of the test results is shown here to provide an overview of the efficacy of the attractant at various volumes and cercariae numbers. The full data table is in S1 Table.

^c^Median values used, full data in S1 Table.

* Indicates values that were calculated rather than extracted directly from the records.

Despite reporting their results in different ways, many of the studies used similar methods. The steps tended to be as follows: (i) preparing the water, (ii) inclusion of the attractant, (iii) introduction of cercariae, (iv) allowing free movement for a fixed exposure period, (v) removal of the trap or collection of cercariae, (vi) quantification of cercariae.

In eight of the eleven studies, light was used to encourage cercariae to shed from infected snails [9–11,24,42–45], a common procedure to obtain cercariae, as detailed by Tucker et al. [46]. Of the papers that specified the snail species, at least 50% used *Biomphalaria glabrata* [9,42,45], the main intermediate host of *S. mansoni* and a widely used snail for laboratory experiments due to its reliability and ease of husbandry [46]. Abellaneda et al. [22] crushed *Oncomelania hupensis quadrasi* snails to obtain *S. japonicum*. Instead of encouraging shedding or crushing snails, Sandt [18] used water from a lighted pool containing cercariae and Klock [12] used natural water samples from a snail breeding area. The details are shown in S2 Table.

Chemical attractants were commonly applied onto slides, which were individually submerged if a smaller water volume was used, such as in a petri dish, or slotted into a stand which was then placed into the water [10,22,24,42,43,45]. Whilst Shiff & Graczyk [11] also applied the attractant on a slide, they chose to use compartments to measure cercarial attraction, with the cercariae placed on the opposite side of the tank to the attractant slide. The tank was then compartmentalised into 14 isolated sections using glass slides after a given amount of time.

The light and temperature experiments had much greater variation. Klock [12] created a more complicated device in which an opaque box containing baffles was lit from above and fed water through an inflow nozzle, whilst Sandt [18] transferred cercariae from a lighted pool to an opaque tube at varying time points to alter the light intensity. Both studies were conducted under a single light condition. Cohen, Neimark & Eveland [9] used a U-tube with heat applied to one end to change the thermal gradient.

Quantification of cercariae was done in all cases using a microscope. In six of the eight chemical studies [10,22,24,42,43,45], after the allotted time for free movement had passed, the slides were removed, allowed to dry, and stained with haematoxylin or iodine. These were then placed under a microscope through which the number of cercariae was counted. One used a T-chamber, placing attractants in each side of the top of the T and counting the number of cercariae that were in each side [44]. In the remaining chemical study, Shiff & Graczyk [11] filtered the water of each of the 14 compartments through individual cellulose acetate membranes, which were then dried and stained with haematoxylin before being examined through a microscope. In a broadly similar manner, Cohen, Neimark & Eveland [9] removed and filtered aliquots, stained the filter, and counted the cercariae using a microscope. Conversely, Klock [12] used a binocular dissecting microscope attached to the flow-controlled chamber to count the cercariae.

The amount of information given by each paper also varied, with some providing many details such as water volume, number of cercariae, water temperature, and attractant strength, and some providing very little experimental information at all. Between papers, there was also a variety of units and recording methods used that made direct quantitative comparison unfeasible. The experimental information provided and the units used by each paper are shown in S4 Text.

The quality of these studies has been rated using an evidence level system. The definition of this system can be found in S3 Text. The level of evidence provided in the studies was spread across the whole range, as two of the eleven studies [10,24] had the highest evidence level of I and three [12,18,44] had the lowest evidence level of VII. Moreover, only three studies [10,12,24] tested the attractants in field-based settings, highlighting this as an aspect where further research may be required. See Table 3.

**Table 3:** Experiment quality of the included studies.

Paper	Evidence Level*
Klock (1961) [12]	VII
Sandt (1972) [18]	VII
Cohen, Neimark & Eveland (1980) [9]	II
Shiff et al. (1993) [24]	I
Shiff & Graczyk (1994) [11]	II
Ahmed et al. (2002) [10]	I
Allah & Ahmed (2011) [42]	VI
Lee et al. (2013) [43]	II
Nobuo (2013) [44]	VII
Lee (2017) [45]	II
Abellaneda et al. (2024) [22]	II

* Definitions can be found in S3 Text.

Only one of the studies created a trap that used two attractant types, chemotaxis and thermotaxis [45]. As well as investigating the effects of beeswax and oleic acid, Lee [45] designed and tested an Environmental Surveillance Device for Schistosome Cercariae (ESDSC) which included beeswax, oleic acid, and a “hand-warmer” to provide heat. The device was tested in a 114-litre aquarium fitted with a ramp at 24° to replicate a shoreline embankment, reducing the water volume to 56.7 litres. 3000 *S. mansoni* cercariae were used for the tests to assess effective ESDSC distance, orientation and depth. Lee [45] found the highest median cercarial capture percentage to be 3.3% when the device was horizontal, near the cercariae origin, and submerged in the water. The device was low-cost (less than $10), required almost no maintenance, used easily sourced materials, required only microscope training, was not labour-intensive and provided rapid results [45]. However, the ESDSC had a low sensitivity, leading Lee [45] to conclude that it would be more appropriate for use as a device for assessing the presence/absence of cercariae rather than quantifying cercariae concentration. Moreover, this trap was not tested in a field-based setting, and Lee [45] suggests that additional data collection and the use of higher temperature “hand-warmers” may allow for device optimisation.

## Discussion

This systematic review has compared and highlighted the variability of the efficacy of attractants for schistosome cercariae. *Schistosoma* cercariae have three major orientation mechanisms: phototaxis, thermotaxis and chemotaxis. The limited number of studies included would suggest that *S. mansoni* are more photo- and thermo-sensitive than chemo-sensitive, despite some literature suggesting otherwise [47,48]. However, direct comparison is not possible due to the varied factors of the experimental methodologies used, such as the water volume and number of cercariae, which have an impact on the efficacy reported. For example, Klock [12] used a complete recovery device with an intake nozzle, baffles to slow water motion and multiple light sources, and Sandt [18] took samples from the top and bottom of the water at various time points. These methodologies are very different to the others, which tended to use simpler slide setups, and both of these studies were also rated in the lowest level of evidence category. This may contribute to the large heterogeneity in efficacy reported. This demonstrates the need for a consistent methodology to allow fair comparison of the efficacy of light, heat, and chemical attractants.

Since the most recent study on light attractants in 1972 [18], there has been development in artificial lighting, including the development of waterproof battery- and solar-powered LEDs. These LEDs could provide an energy-efficient, long-lasting, waterproof, and inexpensive alternative to the mains or generator-powered lighting used by Sandt [18] and Klock [12]. In fact, LEDs are reported to be 75% more energy-efficient than their halogen counterparts and last 30 times longer than an incandescent bulb [49]. LEDs are also relatively small, thus allowing easier incorporation into a range of possible trap designs. Hence, studies on light as an attractant could be further developed to include the use of these LEDs at different wavelengths, voltages, and wattages. This may provide a more appropriate attractant for use in less developed areas with little to no mains power source.

Furthermore, this review found only one paper studying *S. haematobium* and only two studying *S. japonicum*, with the remaining studies focusing on *S. mansoni*. This shows an imbalance in the species studied, likely due to the geographical spread and prevalence of each species [50,51]. The ease of acquiring each species for laboratory use may also be a contributing factor. *Biomphalaria glabrata*, the host snail of *S. mansoni*, is the most commonly kept in laboratory environments, takes the least time from miracidia infection to cercariae release, and has a cercarial production more than four times higher than the other host snails [46]. Also, to obtain *S. japonicum, Oncomelania Hupensis* snails must be crushed, meaning they can be used for cercariae collection only once [46]. The studies that examined *S. japonicum* demonstrated different results to those investigating *S. mansoni* and to those investigating *S. haematobium*. This limited evidence suggests that different cercariae species react differently to different attractants. This is also commented on in existing literature where *S. mansoni* is reported to be more chemokinetic, *S. haematobium* to be more photokinetic, and *S. japonicum* to have little response to stimuli [48].

Additionally, only three of the eleven studies tested attractants in field-based settings [10,12,24], emphasising the lack of progress from laboratory to an actual field-deployable trap that can be inserted into natural water bodies. While some studies concluded that their laboratory-tested attractants could be appropriate for field use [10,12,22,42,43,45], the majority of the studies did not create a complete trap device for testing the capture efficacy in a field-based setting, such as the one developed by Lee [45]. The trap developed by Lee [45] utilised a “hand-warmer”, beeswax, and oleic acid, to exploit both the thermotactic and chemotactic response of *S. mansoni* cercariae. Combining attractant mechanisms may optimise trap performance, improve reliability and hence, increase its adoption potential as a robust environmental surveillance method. Progression from laboratory-based studies of attractants to a field-deployable trap will require consideration of material and production cost, the accessibility of materials, and manufacturing feasibility, all of which will influence its scalability and deployment potential. Beyond trap construction, its locational placement – demonstrated by tests at different depths, distances, and bank angles [45] – the ease of cercarial counting, the level of training and personnel, its operational costs, and community education will be critical to sustained use and meaningful data being obtained. Environmental factors, such as turbidity, vegetation, and diatoms, may also affect the effectiveness of attractants, particularly where water movement, dilution and degradation of chemical cues may reduce the formation and persistence of effective concentration gradients under natural conditions. Hence, these should also be taken into account when designing a trap for field use. Ahmed et al. [10] specified that cercariae were easy to detect even in turbid field conditions. Similarly, Shiff et al. [24] noted that in a turbid field experiment, with many diatoms and seeds present, the linoleic acid still had a much greater efficacy than the control. These two studies [10,24] noted that they had only tested their trap in standing water, and of these, one [10] stated that they planned to carry out future tests in lotic systems. This will provide clearer insight into the effects of water speed on attractant efficacy. The third study to undertake a field-based experiment tested a light apparatus [12]. Whilst no clear information was given on the environmental conditions, this apparatus relied on water flow, and so it may be more appropriate for lotic systems. Consequently, further research and design are required to convert the results of the laboratory-based studies into the development of an affordable, sensitive, and robust cercarial capture device.

Although 1951 records were initially retrieved, only eleven studies met the inclusion criteria. The limited number of eligible studies highlights the underdevelopment of schistosome cercariae traps and attractants.

Despite the comprehensive search strategy used in this systematic review, some relevant studies may have been missed due to inconsistent or non-standardised terminology used to describe *Schistosoma* cercariae attraction methods. Similarly, some studies may have been missed if they were not contained in the databases listed. Whilst two independent screeners were used to screen the papers, only one performed the data extraction, in line with PRISMA systematic review guidance [52].

## Conclusions

This systematic review has demonstrated that there is a limited and methodologically heterogeneous body of evidence on *Schistosoma* cercariae attractants and traps. The mapped evidence suggests that light-based attractants appear promising, although studies are few, heterogeneous, and predominantly laboratory-based. Nevertheless, it is important to note that only one study tested heat and only two tested light, with the two light studies recording the lowest level of evidence rating. *S. japonicum* was only tested in two studies using chemical attractants, to which they were highly responsive, highlighting the potential differences between the two species. Only one study was found on *S. haematobium,* showing a lack of diversity in the species studied. Further limitations of the studies include the inconsistent methodologies and the weakness of evidence of some studies, which impairs direct comparison and limits the ability to make any conclusions on the attractants’ efficacies, and the low numbers of field-based studies. Thus, future research should prioritise standardised protocols and include field-based studies to progress the development of an effective environmental surveillance device to complement human infection data and support schistosomiasis elimination efforts.

## Supporting information

S1 Text Search categories, search terms, and database-specific Boolean search strategies used in the systematic review. (PDF)

S2 Text Inclusion and exclusion criteria. (PDF)

S3 Text Evidence level definitions. (PDF)

S4 Text Experimental parameters and outcome measures reported in included studies. (PDF)

S1 PRISMA Checklist PRISMA checklist. - From: Page MJ, McKenzie JE, Bossuyt PM, Boutron I, Hoffmann TC, Mulrow CD, et al. The PRISMA 2020 statement: an updated guideline for reporting systematic reviews. BMJ 2021;372:n71. doi: 10.1136/bmj.n71. This work is licensed under CC BY 4.0. To view a copy of this license, visit https://creativecommons.org/licenses/by/4.0/.
(DOCX)

S2 PRISMA Checklist PRISMA abstract checklist. From: Page MJ, McKenzie JE, Bossuyt PM, Boutron I, Hoffmann TC, Mulrow CD, et al. The PRISMA 2020 statement: an updated guideline for reporting systematic reviews. BMJ 2021;372:n71. doi: 10.1136/bmj.n71. This work is licensed under CC BY 4.0. To view a copy of this license, visit https://creativecommons.org/licenses/by/4.0/.
(DOCX)

S1 Table Extracted study characteristics and outcome data for all included studies. (XLSX)

S2 Table Attractant type, cercariae source, and snail species reported in included studies. (XLSX)

S3 Table Papers excluded at full-text screening stage. (XLSX)
