# Supplementary material for: Detection of schistosome cercariae in water: A systematic review of traps and attractants
Source: PLoS Negl Trop Dis. 2026 Aug 17;20(8):e0014625. doi: 10.1371/journal.pntd.0014625 (PMC13480631; doi:10.1371/journal.pntd.0014625)
Supplement: Supplementary file 1 [file pntd.0014625.s001.pdf]

S1 Text: Search categories, search terms, and database-specific Boolean search strategies used in the systematic review

Search categories: (schistosomiasis) AND cercariae AND (traps and attractants) AND (measurement methods)

**Search Categories**	schistosomiasis	cercariae	traps and attractants	measurement methods
**Search Terms**	schistosomiasis<br>bilharzia	cercariae	trap<br>attractant<br>bait<br>chemical cue<br>host cue<br>lure	detection<br>monitoring<br>quantification<br>surveillance<br>sampling<br>collection

Google Scholar: (Schistosoma OR schistosomiasis OR bilharzia) AND cercariae AND (trap OR attractant OR bait OR "chemical cue" OR "host cue" OR lure) AND (detection OR monitoring OR quantification OR surveillance OR sampling OR collection)

PubMed: (Schistosoma[tiab] OR bilharzia[tiab]) AND (cercaria*[tiab]) AND (trap*[tiab] OR attract*[tiab] OR bait*[tiab] OR "chemical cue"[tiab] OR "host cue"[tiab] OR lure*[tiab]) AND (detect*[tiab] OR monitor*[tiab] OR quantif*[tiab] OR surve*[tiab] OR sample*[tiab] OR collect*[tiab])

Web of Science: TS=((Schistosom* OR bilharz*) AND (cercaria*) AND (trap* OR attract* OR bait* OR "chemical cue*" OR "host cue*" OR lure*) AND (detect* OR monitor* OR quantif* OR surve* OR sample* OR collect*))

Scopus: TITLE-ABS-KEY((schistosom* OR bilharz*) AND cercaria* AND (trap* OR attract* OR bait* OR "chemical cue*" OR "host cue*" OR lure*) AND (detect* OR monitor* OR quantif* OR surve* OR sample* OR collect*))

Embase: (exp Schistosoma/ or schistosom*.ti,ab. or bilharz*.ti,ab.) and cercaria*.ti,ab. and (trap* or attract* or bait* or "chemical cue*" or "host cue*" or lure*).ti,ab. and (detect* or monitor* or quantif* or surve* or sample* or collect*).ti,ab.

ProQuest: TI((schistosom* OR bilharz*) AND cercaria* AND (trap* OR attract* OR bait* OR "chemical cue*" OR "host cue*" OR lure*) AND (detect* OR monitor* OR quantif* OR surve* OR sample* OR collect*)) OR AB((schistosom* OR bilharz*) AND cercaria* AND (trap* OR attract* OR bait* OR "chemical cue*" OR "host cue*" OR lure*) AND (detect* OR monitor* OR quantif* OR surve* OR sample* OR collect*)) OR SU((schistosom* OR bilharz*) AND cercaria* AND (trap* OR attract* OR bait* OR "chemical cue*" OR "host cue*" OR lure*) AND (detect* OR monitor* OR quantif* OR surve* OR sample* OR collect*))
