# Supplementary material for: Detection of schistosome cercariae in water: A systematic review of traps and attractants
Source: PLoS Negl Trop Dis. 2026 Aug 17;20(8):e0014625. doi: 10.1371/journal.pntd.0014625 (PMC13480631; doi:10.1371/journal.pntd.0014625)
Supplement: Supplementary file 2 [file pntd.0014625.s002.pdf]

S2: Inclusion and Exclusion Criteria

Inclusion	Exclusion
• All languages<br>• Dates from inception until search date<br>• Primary research<br>• Grey literature, such as non-peer-reviewed theses	• Background or commentary articles, not original research<br>• Research relating to cercarial penetration, attachment or creep<br>• Research relating to kinesis rather than taxis<br>• Research relating to non-Schistosome cercariae<br>• Qualitative-only results
