# Supplementary material for: Detection of schistosome cercariae in water: A systematic review of traps and attractants
Source: PLoS Negl Trop Dis. 2026 Aug 17;20(8):e0014625. doi: 10.1371/journal.pntd.0014625 (PMC13480631; doi:10.1371/journal.pntd.0014625)
Supplement: Supplementary file 3 [file pntd.0014625.s003.pdf]

S3 Text: Evidence Level Definitions

**Evidence Level**	**Definition**
**I**	A study that includes all of the following;<br><br>a) Number of cercariae<br><br>b) Species of cercariae<br><br>c) Temperature of water<br><br>d) Size of container<br><br>e) Strength/dosage of attractant (eg lux, g/mL, °C)<br><br>f) Both field and laboratory experiments
II	A study that includes all of the following;<br><br>a) Number of cercariae<br><br>b) Species of cercariae<br><br>c) Temperature of water<br><br>d) Size of container<br><br>e) Strength/dosage of attractant (eg lux, g/mL, °C)<br><br>f) Either field or laboratory experiments
III	A study that includes all of the following;<br><br>a) Number of cercariae<br><br>b) Volume of water<br><br>c) Strength/dosage of attractant (eg lux, g/mL, °C)<br><br>d) Both field and laboratory experiments
IV	A study that includes all of the following;<br><br>a) Number of cercariae

	b) Volume of water<br><br>c) Strength/dosage of attractant (eg lux, g/mL, °C)<br><br>d) Either field or laboratory experiments
V	A study that includes all of the following;<br><br>a) Number of cercariae<br><br>b) Type of attractant<br><br>c) Both field and laboratory experiments
VI	A study that includes all of the following;<br><br>a) Number of cercariae<br><br>b) Type of attractant<br><br>c) Either field or laboratory experiments
VII	A study that doesn't fit any of the above criteria
