# Supplementary material for: Detection of schistosome cercariae in water: A systematic review of traps and attractants
Source: PLoS Negl Trop Dis. 2026 Aug 17;20(8):e0014625. doi: 10.1371/journal.pntd.0014625 (PMC13480631; doi:10.1371/journal.pntd.0014625)
Supplement: Supplementary file 4 [file pntd.0014625.s004.pdf]

S4 Text: Experimental parameters and outcome measures reported in included studies

**Paper**	**Water volume**	**Number of cercariae**	**Cercariae species**	**Attractant type**	**Attractant strength units**	**Duration**	**Measurement units**
Klock (1961) [12]	2000L and a 'large pool'	1	*mansoni*	light	600W and 110V	Unclear in 2000L, 45 minutes in 'large pool'	cercariae capture %
Sandt (1972) [31]	65ml[a]	Not reported	*mansoni*	light	Not reported	20 minutes	cercariae capture %
Cohen, Neimark & Eveland (1980) [9]	32ml	50,000-75,000	*mansoni*	thermal	26 – 34 °C	120 minutes	cercariae per ml
Shiff et al. (1993) [37]	0.05L – 500L[b]	50 – 60,000	*mansoni*	chemical	5μl	1 – 3 hours	cercariae per slide
Shiff & Graczyk (1994) [11]	0.28L[a]	1000	*mansoni*	chemical	5μl	1 – 2 hours	cercariae per compartment
Ahmed et al. (2002) [10]	0.5L[b]-2100L[b]	600 – 42,000	*mansoni*	chemical	Not reported	2 – 3 hours	cercariae per slide
Allah & Ahmed (2011) [39]	Petri dish, 80mm diameter	401	not specified	chemical	1:1 ratio of wax to sesame oil	'overnight'	cercariae per slide
Lee et al. (2013) [40]	20ml	30 – 60	*mansoni*	chemical	0.3 – 1.8g/mL	2 hours	cercariae capture %
Nobuo (2013) [41]	Not reported	Not reported	*mansoni*	chemical	1pM – 10mM	Not reported	cercariae per section
Lee (2017) [42]	20ml	30 – 3000	*mansoni and japonicum*	chemical	0.15 – 0.9g/mL	2 hours	cercariae capture %
Abellaneda et al. (2024) (25)	Petri dish, 70mm diameter	100	*japonicum*	chemical	0.52g/mL olive oil, 250μM pifithrin-μ	2 hours	cercariae capture %

[a] volume calculated using dimensions of containers
[b] volume calculated by comparing cercariae/litre to the number of cercariae used
